# Encapsidation of Staufen-2 Enhances Infectivity of HIV-1

**DOI:** 10.3390/v13122459

**Published:** 2021-12-08

**Authors:** Kannan Balakrishnan, Ananda Ayyappan Jaguva Vasudevan, Krishnaveni Mohareer, Tom Luedde, Carsten Münk, Sharmistha Banerjee

**Affiliations:** 1 Department of Biochemistry, School of Life Sciences, University of Hyderabad, Gachibowli, Hyderabad 500046, India; bkannan7690@gmail.com (K.B.); krishnaveni.mohareer@gmail.com (K.M.); 2 Clinic for Gastroenterology, Hepatology, and Infectiology, Medical Faculty, Heinrich-Heine-University Düsseldorf, 40225 Düsseldorf, Germany; anand.jaguvavasudevan@nih.gov (A.A.J.V.); tom.luedde@med.uni-duesseldorf.de (T.L.)

**Keywords:** Staufen-2, HIV-1 Gag, virus-host interaction, ribonucleoprotein complexes (RNPs), Human Immunodeficiency Virus (HIV), viral incorporation

## Abstract

Staufen, the RNA-binding family of proteins, affects various steps in the Human Immuno-Deficiency Virus (HIV-1) replication cycle. While our previous study established Staufen-2–HIV-1 Rev interaction and its role in augmenting nucleocytoplasmic export of RRE-containing viral RNA, viral incorporation of Staufen-2 and its effect on viral propagation were unknown. Here, we report that Staufen-2 interacts with HIV-1 Gag and is incorporated into virions and that encapsidated Staufen-2 boosted viral infectivity. Further, Staufen-2 gets co-packaged into virions, possibly by interacting with host factors Staufen-1 or antiviral protein APOBEC3G, which resulted in different outcomes on the infectivity of Staufen-2-encapsidated virions. These observations suggest that encapsidated host factors influence viral population dynamics and infectivity. With the explicit identification of the incorporation of Staufen proteins into HIV-1 and other retroviruses, such as Simian Immunodeficiency Virus (SIV), we propose that packaging of RNA binding proteins, such as Staufen, in budding virions of retroviruses is probably a general phenomenon that can drive or impact the viral population dynamics, infectivity, and evolution.

## 1. Introduction

HIV-1 relies on host cellular machinery for its infectivity and propagation. HIV-1 RNA associates with many host RNA binding proteins (RBPs) during its transport and translation. The molecular composition of HIV-1 RNA-associated RBP containing ribonucleoprotein (RNP) complexes undergo dynamic changes during virus propagation, associating with distinct host proteins at different stages, such as splicing, nuclear export of viral RNA, translation, assembly, and budding [1,2,3]. The cumulative impact of these interactions determines HIV-1 production and viral population dynamics.

HIV-1 Gag plays a crucial role in viral RNA encapsidation and particle assembly. During this process, HIV-1 Gag associates with several host RBPs forming RNP complexes. The host proteins bound to HIV-1 Gag and viral RNA in RNPs are often packed into new virus particles [4,5]. Gag-interacting host factors can either support or restrict viral replication. One such example is a restriction factor APOBEC3G (A3G), which interacts with the nucleocapsid (NC) domain of HIV-1 Gag and gets packaged into progeny virions in the producer cells in the absence of HIV-1 Vif protein that degrades A3G. During the next round of infection, the incorporated A3G binds to the minus strand of the proviral DNA and generates mutations by cytidine deamination, thereby inhibiting HIV-1 [6,7,8,9,10]. Similarly, Staufen-1 interacts with the NC domain of HIV-1 Gag and is incorporated into emerging virus particles, usually acting as a positive regulator of HIV-1 propagation [11,12,13,14].

Staufen is a family of RBPs that affects RNA localization in many organisms [15]. First identified in Drosophila, Staufen proteins were later found to be ubiquitously expressed in all mammalian cells and are known to play diverse roles in mRNA transport, translation, and mRNA decay [15,16,17]. Mammalian cells express two Staufen paralogs termed Staufen-1 and Staufen-2; both proteins are derived from separate genes and produce several isoforms via pre-mRNA alternative splicing or polyadenylation. Both Staufen-1 and Staufen-2 contain four double-stranded RNA-binding domains (dsRBD) and bind to distinct but overlapping sets of mRNAs [18,19]. Although several studies define multiple roles of Staufens in neural cells [20,21,22,23], these proteins are also expressed across different cell types, including T cells and macrophages, and are involved in different functions, such as cell-cycle regulation, apoptosis, organogenesis, and regulation of inflammatory and immune response genes expression [18,24,25,26,27,28,29].

While both Staufen proteins have been studied extensively for their roles in regulating the post-transcriptional activity of RNA transport, translation, and decay, Staufen-1 has been recognized as a positive effector of several RNA viruses, such as enterovirus [30], influenza virus [31], hepatitis C virus [32], HCV [33], endogenous HERV-K (HML-2) [34], and HIV-1 [12,35]. Staufen-1 interacts with HIV-1 Gag and HIV-1 RNA to facilitate various activities such as RNA transport and packaging [35,36]. Like Staufen-1, Staufen-2 aids in the trafficking of RNP granules in neurons and is actively involved in mRNA transport and local translation [37,38,39]. Interaction of Staufen-1 and Staufen-2 with HIV-1 RNA was reported to enhance viral RNA encapsidation [14]. Aside from interacting with HIV-1 RNA, our previous study established that Staufen-2 interacts with HIV-1 protein Rev and increases Rev-associated viral RNA export from the nucleus to the cytoplasm [40]. A similar association of Staufen-1 with HIV-1 Rev is not reported, although Staufen-1 has been shown to interact with Rec of HERV-K (HML-2) [34]. Interestingly, Staufen-1 and Staufen-2 are reported to co-localize in cells and occur as typical components of RNP granules [18,39]. Considering the involvement of Staufen-1 in supporting HIV-1 infection, we set out to investigate if Staufen-2 gets encapsidated into virus particles and affects viral infectivity.

## 2. Materials and Methods

### 2.1. Cell Culture

Wild type HEK293T cells (WT) and its Staufen knockout cell lines were maintained in Dulbecco’s high-glucose Modified Eagle’s Medium (DMEM) (Biochrom, Berlin, Germany), supplemented with 10% FBS (Biowest, Nuaillé, France), 2 mM L-glutamine, 50 units/mL penicillin, and 50 µg/mL streptomycin (Gibco, Schwerte, Germany) at 37 °C in a humidified atmosphere of 5% CO_2_.

### 2.2. Plasmids

pCMV-sport1 Staufen-2 59 FLAG was used as a template to generate the Staufen-2 stable cell line. Staufen-2 was amplified and cloned into SpeI and NotI sites of the pLVX-IRES-Puro vector with N-terminal FLAG using forward primer 5′ACTACTAGTACCATGGACTACAAGGACGACGACGACAAAATGCTTCAAATAAATCAG3′ and reverse primer: 5′ACTGCGGCCGCCTAGACGGCCGAGTTTGATT3′. pCMV-sport1 Staufen-2 59 FLAG and pcDNA3-RSV-Stau1 55 HA expression plasmids were kind gifts from Luc Desgroseillers [11]. Nathaniel R. Landau generously provided an A3G HA expression vector. Myc-6His and V5 tagged A3G (pcDNA3.1 expressing human A3G Myc-6His and A3G V5) were described previously [41]. pcDNA5-Staufen-1 FLAG vector was generously provided by Jernej Ule [42]. pkGST-HIV-1 Gag was a gift from Bryan Cullen [43]. LentiCRISPRv2 targeting Staufen-2 and Staufen-1 genes were constructed according to previously described protocols [44,45]. Single guide RNAs (sgRNAs) targeting Staufen-2 forward primer 5′CACCGAGAGCTAATTACAACTTTCG3′ and reverse primer 5′AAACCGAAAGTTGTAATTAGCTCTC3′, and of Staufen-1 sgRNA forward primer 5′CACCGGAAAACAAAACCCATAGTCA3′ and reverse primer 5′AAACTGACTATGGGTTTTGTTTTCC3′ with BsmBI overhang were annealed and ligated into pLentiCRISPRv2.

### 2.3. Virus Production and Isolation

HEK293T cells were transiently transfected using Lipofectamine LTX and Plus reagent (Invitrogen, Karlsruhe, Germany) with an appropriate combination of HIV-1 viral vectors 1800 ng of NL-Luc R^−^ E^−^ [46], 250 ng pRSV-Rev, 150 ng pMD.G, or 600 ng pMDLg/pRRE (HIV-1 packaging plasmid) [47], 600 ng pSIN.PPT.CMV.Luc.IRES.GFP (expresses the firefly luciferase and GFP), 250 ng pRSV-Rev, and 150 ng pMD.G (encodes the glycoprotein of VSV (VSV-G) [47] with 800 ng of either Staufen-2 FLAG/A3G-HA/Staufen-1 FLAG alone or in combination as represented, in a 6 well plate. The plasmid DNA concentrations were normalized with pcDNA3.1. NL-Luc R^−^ E^−^ plasmids were transfected into HEK293T vector cells, Staufen-2 knockout cells and Staufen-1 knockout cells to evaluate viral infectivity in the respective cells. Replication-competent, infectious HIV-1 particles were produced from HEK293T cells by transfection with 1500 ng of proviral DNA pNL4-3 [48], 800 ng Staufen-2 FLAG, or 800 ng Staufen-1 FLAG (as titrated to achieve optimal relative infectivity) and normalized by pcDNA3.1. After 48 h of transfection, virion-containing supernatants were collected. For the isolation of virions, the virion containing supernatant was concentrated by layering on a 20% sucrose cushion and centrifuged for 4 h at 14,800 rpm, 4 °C. Viral particles were resuspended in mild lysis buffer containing 50 mM Tris (pH 8), 1 mM PMSF, 10% glycerol, 0.8% NP-40, 150 mM NaCl, and 1X complete protease inhibitor. Simian Immunodeficiency Virus SIV_AGM_TAN-1 (pSIV_AGM_-Luc-R^−^E^−^Δ*vif*) reporter viruses were prepared as described previously [49].

### 2.4. Generation of Stably over Expressing Staufen-2 and Vector HEK293T Cell Lines

HEK293T cells were transfected with 800 ng pLVX-IRES-PURO vector alone or 800 ng pLVX-IRES-PURO-Staufen-2 FLAG with psPAX-based lentiviral packing system. After 72 h of transfection, the viral particles were collected and used for the HEK293T target cell transduction. The cells were selected using 1 μg/mL puromycin for 3 days, and the stable overexpression of Staufen-2 FLAG proteins was assessed by Western analyses using an anti-FLAG antibody.

### 2.5. HIV-1 Viral Core Isolation

Viral cores were isolated as described earlier [50,51]. In brief, HIV-1Δ*vif* viral particles were produced in the presence of Staufen-2 FLAG. After 48 h of transfection, the HIV-1 virions were concentrated by centrifugation through a 20% sucrose cushion in an ultracentrifuge at 136,000 g for 2 h. The concentrated viral particles were suspended in STE buffer containing 10 mM Tris–HCl (pH 7.4), 100 mM NaCl, and 1 mM EDTA [52], layered on the top of the sucrose step gradient containing a layer of either buffer or 1% Triton X-100 (in 15% sucrose), as represented in Supplementary Figure S1, and centrifuged at 136,000 g for 1 h at 4 °C in ultra-clear centrifuge tubes (13 × 51 mm) in an MLS-50 rotor (Beckman Coulter, Fullerton, CA, USA). Three fractions, F1, F2, and F3, were collected from the top of the sucrose step gradient, and an aliquot of each fraction was subjected to Western blot analysis. Viral compartments and the Staufen-2 FLAG proteins were detected by probing the blots with respective antibodies anti-FLAG (Staufen-2 FLAG), anti-p24 (HIV-CA), and anti-VSV-G.

### 2.6. Generation of Staufen-2 and Staufen-1 Knockout HEK293T Cell Lines

Staufen-2 and Staufen-1 knockout HEK293T cells were produced by using CRISPR/Cas9 gene editing. Staufen-2 and Staufen-1 coding sequences were obtained from NCBI, and sgRNAs were designed using the GPP web portal (https://portals.broadinstitute.org/gpp/public/; accessed on 7 November 2017). The top-scoring sgRNA oligos were selected and cloned into LentiCRISPRv2 with BsmBI, overhang. The clones were confirmed by sequencing, and the LentiCRISPRv2 containing the corresponding sgRNA sequence was transfected with a lentiviral packing system in HEK293T cells. After 72 h of transfection, the viral particles were collected and subsequently used to infect HEK293T target cells. The cells were selected by using 1 μg/mL puromycin. CRISPR-knockout of Staufen-2 and Staufen-1 proteins was validated by Western blotting with respective antibodies. Staufen-2 and Staufen-1 knockout single-cell clones were prepared by limiting dilution and screened for the lack of expression of either Staufen-2 or Staufen-1 (Supplementary Figure S2).

### 2.7. Luciferase-Based Infectivity Assay

HIV-1 luciferase reporter viruses were used to infect HEK293T cells and used for the infectivity assay. Before infection, the amount of reverse transcriptase (RT) in the viral particles was determined by RT assay using Cavidi HS kit Lenti RT (CavidiTech, Uppsala, Sweden). As described earlier, an equal amount of RT normalized viral particles were used for viral infection [50,53,54,55]. After 48 h, luciferase activity was measured using SteadyliteHTS luciferase reagent substrate (Perkin Elmer, Rodgau, Germany) in black 96-well plates on a Berthold MicroLumat Plus luminometer (Berthold Detection Systems, Pforzheim, Germany). Infections were performed in triplicate, and at least three independent experiments were performed.

### 2.8. TZM-bl Infectivity Assay

The adherent TZM-bl cells were seeded 24 h prior to infection. Following infection with 15 ng of p24 equivalent of HIV-1 NL4-3 virus in complete DMEM for 6 h, the residual virus was removed, the cells were washed with PBS and incubated in fresh complete DMEM for 48 h. The medium was removed, the cells were washed with PBS and lysed. The cell lysates were quantified for luciferase activity as per the manufacturer’s protocol (Promega, Madison, WI, USA).

### 2.9. Immunoblot Analyses

Transfected HEK293T cells were washed with phosphate-buffered saline (PBS) and lysed in radioimmunoprecipitation assay buffer (RIPA, 25 mM Tris (pH 8.0), 137 mM NaCl, 1% glycerol, 0.1% SDS, 0.5% sodium deoxycholate, 1% Nonidet P-40, 2 mM EDTA, and protease inhibitor cocktail) for 20 min on ice. The lysates were clarified by centrifugation (20 min, 14,800 rpm, 4 °C). Samples (cell/viral lysate) were boiled at 95 °C for 5 min with Roti load reducing loading buffer, subjected to SDS-PAGE, and then transferred to a PVDF membrane. Membranes were blocked with skimmed milk solution and probed with appropriate primary antibodies followed by respective secondary antibodies. Signals were visualized using ECL chemiluminescent reagent (GE Healthcare, Munich, Germany).

### 2.10. Immunoprecipitation

To investigate the protein–protein interactions, expression constructs including empty vector plasmids were transiently transfected into HEK293T cells. After 48 h of transfection, the cells were lysed using mild lysis buffer (50 mM Tris (pH 8), 1 mM PMSF, 10% glycerol, 0.8% NP-40, 150 mM NaCl, and 1X complete protease inhibitor). The clarified lysate was incubated with either anti-HA affinity matrix (A3G or Staufen-1 based immunoprecipitation) or anti-FLAG affinity matrix (Staufen-2 based immunoprecipitation) beads for 4 h at 4 °C, with end-over-end rotation. To check whether the interaction is RNA dependent or independent, the samples were divided into two halves, and one half was treated with RNase A (70 μg/mL) and incubated for 10 min at 37 °C, followed by 40 min at 22 °C [56]. Samples were further washed thrice with lysis buffer. Co-IP products were eluted by boiling beads in SDS gel loading buffer at 95 °C for 5 min. The interactions were analyzed by immunoblotting with respective antibodies.

### 2.11. GST Pull-Down Assay

HEK293T cells were co-transfected with either 1.2 μg of pkGST vector alone (control) or pkGST-HIV-1 Gag and 1.2 μg of Staufen-2 FLAG, or Staufen-1 FLAG construct (positive control). After 48 h of transfection, the cells were lysed using mild lysis buffer (50 mM Tris (pH 8), 1 mM PMSF, 10% glycerol, 0.8% NP-40, 150 mM NaCl, and 1X complete protease inhibitor). The clarified cell lysate was incubated with Glutathione Sepharose 4B beads for 4 h at 4 °C, with end-over-end rotation. After binding, the GST beads were washed three times with mild lysis buffer, and proteins bound to GST beads were eluted by boiling beads in SDS gel loading buffer at 95 °C for 5 min. The interactions were analyzed by immunoblotting with respective antibodies. As described in the immunoprecipitation section, RNase A treatment was performed to study if the protein–protein interaction is RNA-bridged.

### 2.12. Immunofluorescence

HEK293T cells grown on polyethylene coverslips (Thermo Fisher Scientific, Vilnius, Lithuania) were co-transfected with the expression plasmids for Staufen-2 FLAG or c-Flag pcDNA3 with A3G HA or Staufen-1 HA or HIV-1 Gag EGFP using Lipofectamine LTX and Plus reagent (Invitrogen, Karlsruhe, Germany). After 24 h of transfection, cells were fixed with 4% paraformaldehyde in phosphate-buffered saline (PBS) for 10 min, permeabilized with 0.1% Triton X-100 for 10 min and incubated with blocking solution (10% FBS in PBS) for 1 h. Then, cells were stained with rabbit anti-Flag antibody (Sigma-Aldrich, Taufkirchen, Germany) in a 1:1000 dilution in blocking solution for 1 h and mouse anti-HA (Sigma-Aldrich, Taufkirchen, Germany) antibody in a 1:1000 dilution in blocking solution for 1 h. Secondary antibodies, Donkey anti-rabbit Alexa Fluor 594 (Thermo Fisher Scientific, Baltics, UAB) followed by Donkey anti-mouse Alexa Fluor 488 (Covance, Münster, Germany), were used at a 1:300 dilution in blocking solution for 1 h. Finally, DAPI was used to stain nuclei for 2 min. The images were captured using a 60× objective on a Zeiss LSM 510 Meta laser scanning confocal microscope (Carl Zeiss, Cologne, Germany). The images were analyzed by ZEN 2.1 (blue edition) software (Carl Zeiss).

### 2.13. Sucrose Density Gradient Centrifugation

HEK293T cells were transfected with 1 μg of Staufen-2 alone or co-transfected with 1 μg of A3G HA or HIV-1 proviral constructs. After 36 h of transfection, the cells were lysed with HMM complex lysis buffer (10 mM Tris (pH 7.4), 100 mM NaCl, 50 mM potassium acetate, 10 mM EDTA, 0.7% NP-40), and the samples were clarified by centrifugation for 10 min at 162 g followed by a short spin at 18,000 g for 30 s. One half of the sample was used to determine the effect of RNase A treatment with 70 μg/mL RNase A for 30 min at 37 °C. The samples were overlaid on the top of 10%–15%–20%–30%–50% sucrose step gradient in HMM lysis buffer and centrifuged for 45 min at 163,000 g at 4 °C in an MLS-50 rotor as described before [50]. After centrifugation, the samples were sequentially removed from the gradient, resolved by SDS-PAGE, and analyzed by Western blotting.

### 2.14. In Vitro DNA Cytidine Deamination Assay

HEK293T cells were transfected with vector alone or A3G V5, A3G V5 with increasing concentration of Staufen-2 FLAG expression plasmid. After 48 h of transfection, the cells were lysed using mild lysis buffer (50 mM Tris (pH 8), 1 mM PMSF, 10% glycerol, 0.8% NP-40, 150 mM NaCl, and 1X complete protease inhibitor) and used for in vitro DNA cytidine deamination assay as described earlier [53,56]. Above mentioned protein samples were added in the cytidine deamination reactions in a 10 µL reaction volume containing 25 mM Tris pH 7.0, 2 µL of cell lysate or respective concentration of purified protein, and 100 fmol of known single-stranded DNA (ssDNA) substrate for A3G (CCCA) 5′GGATTGGTTGGTTATTTGTTTAAGGAAGGTGGATTAAAGGCCCAAGAAGGTGATGGAAGTTATGTTTGGTAGATTGATGG-3′. Reaction mixtures were treated with 50 µg/mL RNase A (Thermo Fisher Scientific, Baltics, UAB). Samples were incubated for 1 h at 37 °C, and the deamination reaction was terminated by boiling at 95 °C for 5 min. One fmol of the reaction mixture was used for PCR amplification with Dream Taq polymerase (Thermo Fisher Scientific, Baltics, UAB) (95 °C for 3 min, followed by 30 cycles of 61 °C for 30 s and 94 °C for 30 s) using specific primers: forward primer, 5′-GGATTGGTTGGTTATTTGTTTAAGGA-3′; reverse primer, 5′CCATCAATCTACCAAACATAACTTCCA-3′. PCR products were digested with Eco147I (StuI) restriction enzyme and the samples were resolved on 15% native PAGE, stained with ethidium bromide (7.5 μg/mL). Instead of the CCA oligonucleotide substrate, CCU was used as a positive control for the Eco147I (StuI) digestion.

### 2.15. A3G Degradation Assay

HEK293T cells were transfected with A3G HA or HIV-1 Vif V5 or A3G HA with HIV-1 Vif V5 expression plasmid. After 48 h of transfection, the cells were lysed using standard RIPA buffer supplemented with 1X complete protease inhibitor and used for Western blot analyses. Similarly, HEK293T vector cells and HEK293T Staufen-2 KO cells were transfected with respective plasmids. After 48 h of transfection, the cells were lysed using RIPA buffer for Western blot analyses.

### 2.16. Statistical Analysis

Statistical analyses were performed using GraphPad Prism version 8.0.2 (GraphPad Software, Inc., La Jolla, CA, USA), and Image J software was used wherever required. The mean values were plotted, and the standard deviations (SD) from the mean of at least three independent experiments are shown as error bars. The study groups were compared using a two-tailed, unpaired Student *t*-test, and a *p*-value of <0.05 was considered statistically significant.

## 3. Results

### 3.1. Staufen-2 Is Encapsidated into HIV-1 Virions, Enhancing Their Infectivity

**Figure 1:**
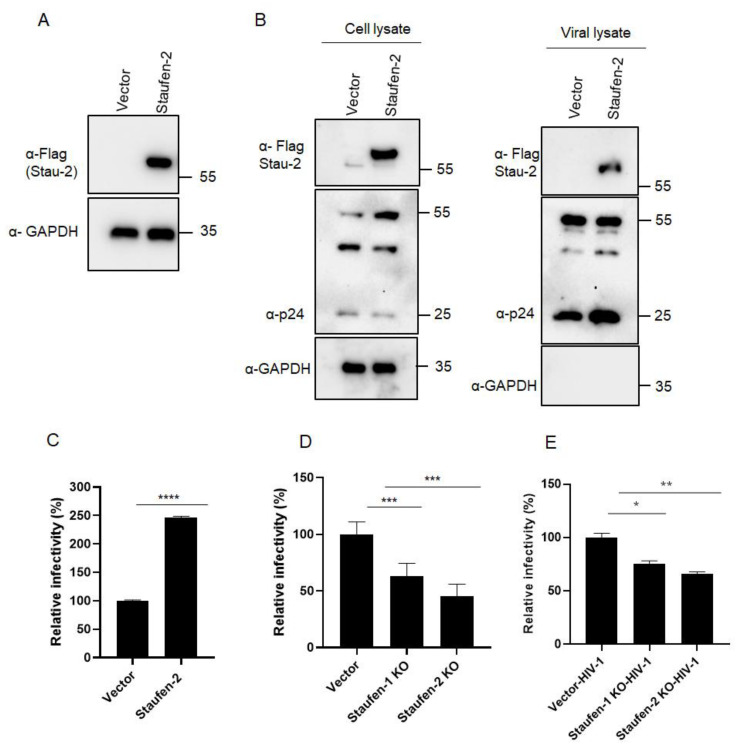
Staufen-2 is incorporated into HIV-1, enhancing viral infectivity: (**A**) Staufen-2 stable cell line was generated by transfection of HEK293T cells with pLVX-IRES-PURO Vector alone (vector cells) or pLVX-IRES-PURO-Staufen-2 FLAG and selected using puromycin. The stable overexpression of Staufen-2 FLAG proteins was confirmed by Western analyses using anti-FLAG antibodies. (**B**) Vector and Staufen-2 stable cell lines were transfected with NL-Luc R^−^ E^−^. After 48 h of transfection, the total cell and viral lysates were prepared. Western analyses were carried out with anti-FLAG (Staufen-2) and anti-p24 antibodies (HIV-1 capsid protein) in the cell viral lysate. GAPDH served as a loading control in both A and B. (**C**) NL-Luc R^−^ E^−^ viral particles were produced in vector and Staufen-2 stable cell lines. An equal amount of RT-activity normalized viral particles were used for the luciferase-based infectivity assay in HEK293T cells. Viral infectivity was quantified relative to the virus lacking Staufen-2. The presented values represent mean ± standard deviations (error bars) for three independent experiments. * *p*-value ≤ 0.05 was considered significant. (**D**) NL-Luc R^−^ E^−^ viral particles were used to infect vector cells, Staufen-2 KO, Staufen-1 KO cells, and luciferase-based infectivity assays were performed. Viral infectivity in the Staufen-2 stable cell line was quantified relative to the vector cells and Staufen-1 KO cell line. (**E**) An equal amount of RT-activity normalized NL-Luc R^−^ E^−^ viral particles generated from HEK293T vector, HEK293T Staufen-2 KO, and Staufen-1 KO producer cells were used for the luciferase-based infectivity assay in HEK293T cells. Asterisks (****) indicates *p*-value ≤ 0.0001, (***) indicates *p*-value ≤ 0.0006 and (**) indicates *p*-value ≤ 0.0058.

To investigate if Staufen-2 is packaged into HIV-1 virions and modulates viral infectivity, we generated HEK293T cells that stably expressed Staufen-2 (isoform 59 kDa) with a FLAG tag (Figure 1A). The Staufen-2 stably-expressing cells, as well as vector-integrated (control) cells, were transfected with an expression plasmid for the HIV-1 luciferase reporter virus (NL-LucR^−^ E^−^ along with pMDG.VSV-G) [46]. To assess the level of Staufen-2 expression in cell lysates and the presence of Staufen-2 in viral particles, Western blot analyses were performed 48 h post-transfection (Figure 1B). Staufen-2 was detected in both cell and viral lysates prepared from stably expressing cells and was absent in the control cells (Figure 1B). In addition, we isolated HIV-1 viral cores by sucrose density gradient centrifugation and evaluated the presence of Staufen-2 in these viral compartments (Supplementary Figure S1).

**Figure 2:**
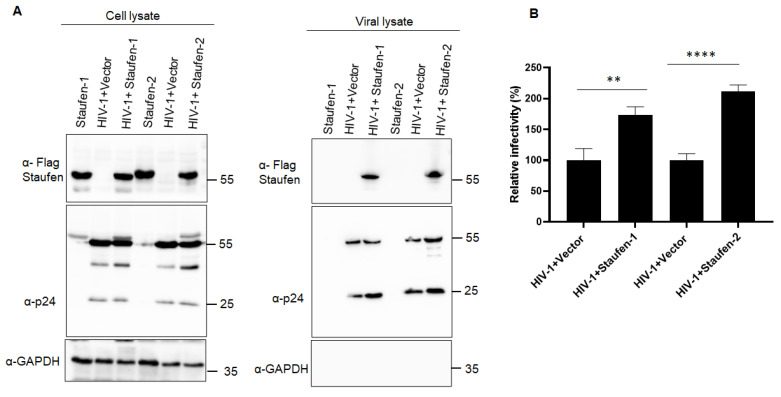
Impact of encapsidated Staufen proteins on replication-competent NL4-3 HIV-1 infectivity: (**A**) NL4.3-based HIV-1 viral particles were produced from HEK293T cells in the presence of Staufen-1 FLAG or Staufen-2 FLAG, or vector alone. (**B**) An equal amount of p24 normalized viral particles were used for the TZM-bl infectivity assay. Viral infectivity was quantified relative to the virus prepared with vector control. The presented values represent mean ± standard deviations (error bars) for three independent experiments. Asterisks (**) indicates *p*-value ≤ 0.0026 and (****) indicates *p*-value ≤ 0.0001.

Next, we evaluated the impact of Staufen-2 packaging on viral infectivity using a luciferase-based infectivity assay. HIV-1 NL-LucR^−^ E^−^ viral particles were produced as VSV-G pseudotypes in HEK293T vector and Staufen-2 stable cell lines. An equal amount of RT-activity normalized viral particles were used to infect HEK293T cells, and the luciferase activity was determined 48 h later. Virions of Staufen-2 cells had a 2.5-fold enhancement of their infectivity, suggesting a positive influence of Staufen-2 on viral infectivity (Figure 1C). To further characterize the role of Staufen-2 in HIV-1 infectivity, we generated HEK293T-Staufen-2 knockout (KO) cells (Supplementary Figure S2A,B) as well as Staufen-1 KO cells as a control (Supplementary Figure S2C,D). As a lower viral infectivity phenotype was reported in the Staufen-1 KO cell [12,35], we performed a side-by-side comparison using Staufen-2 and Staufen-1 KO cells infected with HIV-1 Luc reporter viruses. It was observed that while both reduced viral infectivity, Staufen-2 KO background reduced the viral infectivity by 55%, while Staufen-1 KO background reduced the viral infectivity by 33%. Thus, the reduction in the viral infectivity was marginally more in Staufen-2 KO than Staufen-1 KO (Figure 1D). HEK293T cells were then infected with normalized virions produced from WT and KO cell lines. Virions generated from Staufen-1 KO and Staufen-2 KO cells showed a 25% and 34% reduction in infectivity, respectively, compared to virions from WT cells (Figure 1E). Similar results were observed in an infectivity assay using TZM-bl reporter cells infected with replication-competent NL4.3 HIV-1 virions containing Staufen proteins (Figure 2). Together, the data indicate a possible impact of the packaged Staufen-2 on viral infectivity.

### 3.2. Staufen-2 Is Packaged into HIV-1 through Its Interaction with Gag

**Figure 3:**
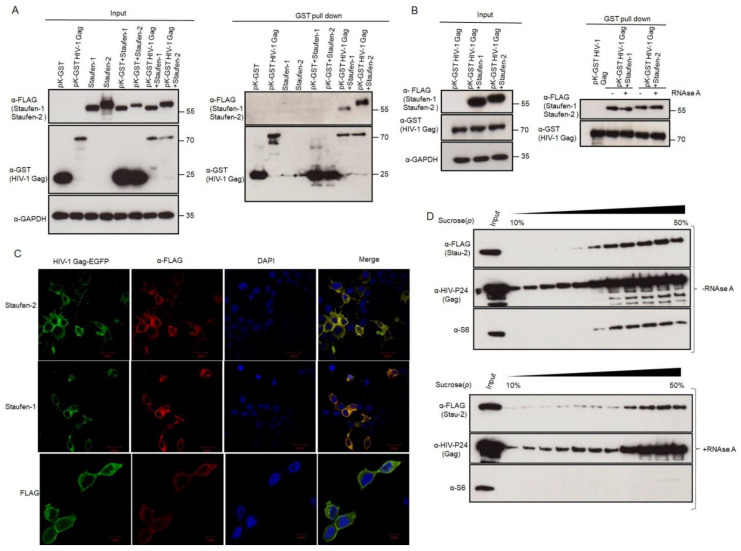
Staufen-2 interacts with HIV-1 Gag: (**A**) HEK293T cells were co-transfected with either pkGST or pkGST-HIV-1 Gag and Staufen-2 FLAG or Staufen-1 FLAG (positive control) constructs. The overexpression of proteins was confirmed by Western analyses (input). After 48 h of transfection, total cell lysates were prepared, and a GST pull-down assay was performed, followed by Western analyses with anti-FLAG (Staufen-2 or Staufen-1) and anti-GST (HIV-1 Gag GST or GST alone) antibodies. (**B**) GST pull-down assay was performed with or without RNase A treatment of the total cell lysates and probed with anti- FLAG (Staufen-2 or Staufen-1) and anti-GST (HIV-1 Gag GST or GST alone) antibodies. GAPDH served as a loading control in both A and B. (**C**) Staufen-2 FLAG, or Staufen-1 FLAG and HIV-1 Gag-EGFP were localized by HIV-1 Gag (EGFP) or Staufen-2 and Staufen-1 (FLAG-TR red). Nuclei are stained by DAPI (in blue). The co-localized complexes are seen as yellow rings in the merged panel. (**D**) The RNP complexes (with or without RNase A treatment) were separated based on their molecular size and density. Each fraction was checked for the presence of the transiently expressed Staufen-2 FLAG and HIV-1 Gag by Western analyses. Ribosomal protein S6 was used as a control.

To investigate if Staufen-2 interacts with HIV-1 Gag for encapsidation into virions, we performed a GST pull-down assay by transfecting expression plasmids Gag-GST and Staufen-2-FLAG. Staufen-1 FLAG was used as a positive control for interaction with HIV-1 Gag. Total cell lysates were extracted 48 h post-transfection, and the GST pull-down products were probed with an anti-FLAG antibody to confirm the interaction between Staufen and HIV-1 Gag (Figure 3A). The immunoblot analyses showed an interaction of both Staufen-1 and -2 with Gag-GST, but no interaction was detected with GST alone (Figure 3A). To evaluate an RNA dependence of the Staufen-2 GAG interaction, we treated the GST pull-down products with RNase A (Figure 3B). The interaction between Staufen-2 and HIV-1 Gag and Staufen-1 and HIV-1 Gag was not disrupted upon RNase A treatment, indicating that both Staufen-1-Gag and Staufen-2-Gag interactions are RNA-independent (Figure 3B).

Additionally, we monitored the localization of both HIV-1 Gag (EGFP tagged) and Staufen-2 (FLAG-tagged) upon co-transfection, followed by confocal microscopy. The co-localization of HIV-1 Gag and Staufen-2 was observed as orange/yellow rings (Figure 3C, upper panel) in the cytoplasm as in the positive control Staufen-1 (Figure 3C, middle panel). Vector control cells (FLAG) transfected with EGFP tagged HIV-1 Gag was used as a negative control, where the merged images clearly showed no overlap (Figure 3C lower panel).

Next, the Staufen-2 over-expressing cells were co-transfected with HIV-1 proviral vector, and RNPs were isolated using sucrose step-density gradient centrifugation after 36 h post-transfection. We observed that Staufen-2 was associated with HIV-1 Gag containing RNPs (Figure 3D). The association of Staufen-2 with these RNPs was not considerably affected in the presence of RNase A, corroborating our immunoprecipitation results, wherein the interaction between Staufen-2 and HIV-1 Gag was found to be RNA-independent. Ribosomal protein S6 was used as an RNP marker, the association of which is known to be RNA dependent [57,58]. These observations suggest that Staufen-2 interacts with HIV-1 Gag and is incorporated into HIV-1 virions.

### 3.3. Multiple Pathways Mediate Staufen-2 Incorporation into Virions

#### 3.3.1. Staufen-2 Interacts with Staufen-1

**Figure 4:**
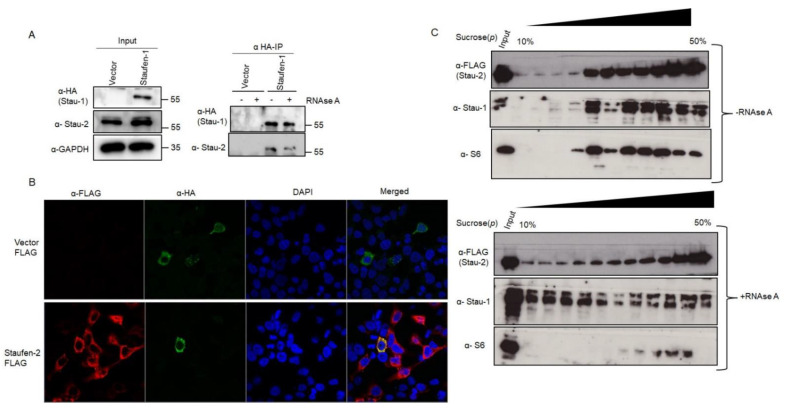
Staufen-2–Staufen-1 interaction is RNA-independent: (**A**) HEK293T cells were transfected with vector alone or with Staufen-1 construct. After 48 h of transfection, total cell lysates were prepared, followed by IP with anti-HA affinity matrix was performed without or with RNase A treatment. The expression of Staufen-1 (anti-HA) and Staufen-2 (anti-Stau-2) were confirmed by Western analyses with GAPDH as a loading control. (**B**) Staufen-2 FLAG (red) or Staufen-1 HA (green) were localized by confocal microscopy. Nuclei were stained by DAPI (in blue). The co-localized complexes are seen as yellow rings in the merged panel. (**C**) The RNP complexes, either with or without RNase A treatment, were separated based on their molecular size and density. Each fraction was monitored for Staufen-2 FLAG and Staufen-1 by Western analyses with ribosomal protein S6 as a control for the RNP complex.

Staufen-1 and Staufen-2 have been reported to interact with each other in vitro [22]. To investigate if these two proteins interact with each other during HIV-1 propagation, we performed immunoprecipitation assays in HEK293T cells transfected with either pcDNA3 or pcDNA3 RSV Stau-1 HA (Staufen-1 with HA tag) using an anti-HA affinity matrix. The pull-down product was probed with an anti-Staufen-2 antibody. Figure 4A shows that Staufen-2 is detectable in Staufen-1 immuno-precipitate independent of treatment with RNase A, supporting an RNA-independent interaction of Staufen1-HA with Staufen-2 (Figure 4A). In addition, we performed a reverse IP, wherein we pulled FLAG-tagged Staufen-2 to investigate if it coprecipitates with HA-tagged Staufen-1 (Supplementary Figure S3A). The interaction between Staufen-1 and Staufen-2 was also confirmed by confocal microscopy, wherein Staufen-1 and Staufen-2 co-localized in the cytoplasm (Figure 4B and Supplementary Figure S3B). Upon investigating the RNPs extracted by sucrose step-gradient centrifugation from HEK293T cells over-expressing Staufen-2, we observed that endogenous Staufen-1 and overexpressed Staufen-2 FLAG co-associated in the same RNP fractions, which was partially affected by RNase A treatment, confirming their interactions under physiological conditions (Figure 4C). With the co-localization in the same RNPs, we additionally observed that both Staufen-1 and Staufen-2 were found in viral lysates from the cells transiently overexpressing these proteins, indicating the possibility of encapsidation of these proteins together in emerging virions (Supplementary Figure S4A), although individual proteins getting differentially packaged within the viral population cannot be ruled out.

#### 3.3.2. Staufen-2 Interacts with APOBEC3G

**Figure 5:**
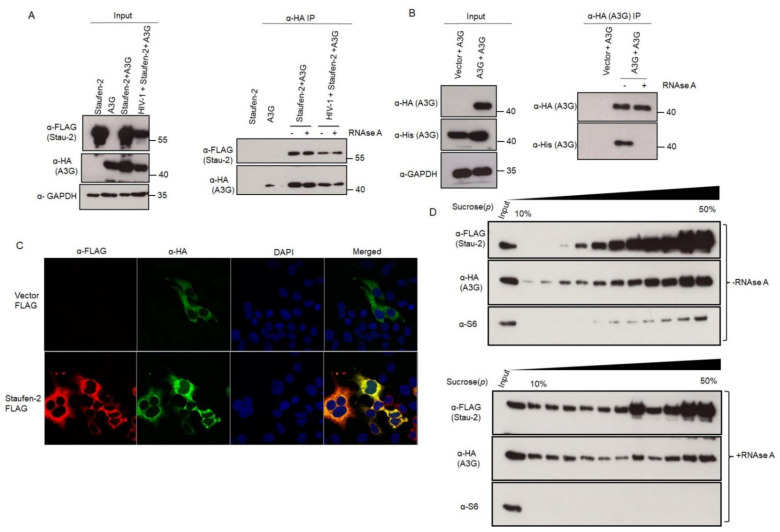
Staufen-2 interacts with antiviral protein A3G: (**A**) HEK293T cells were co-transfected with either vector alone or A3G HA and Staufen-2 FLAG with or without HIV-1 proviral construct. After confirming the expression of individual proteins by Western analyses (input), total cell lysates were prepared 48 h post-transfection, followed by IP with anti-HA affinity matrix without or with RNase A treatment and probed with anti-FLAG (Staufen-2) and anti-HA (A3G) antibodies. (**B**) HEK293T cells were co-transfected with either vector (HA) or A3G HA-tagged and A3GHis-tagged constructs. Total cell lysates were prepared, either with or without RNase A treatment, followed by immunoprecipitation with anti-HA beads and analyzed by Western blotting using anti-His antibodies. GAPDH served as a loading control. (**C**) Staufen-2 FLAG and A3G HA were localized by A3G HA (AF488 Green) or Staufen-2 FLAG (AF594 red). Nuclei were stained by DAPI (in blue). The co-localized complexes are seen as yellow rings in the merged panel. (**D**) The RNP complexes (with or without RNase A treatment) were separated based on their molecular size and density. Each fraction was monitored for the presence of Staufen-2 FLAG and A3G HA by Western analyses. Ribosomal protein S6 served as a control for RNP complexes.

Given the similarity of APOBEC3G (A3G) and Staufen-2 in binding to HIV-1 Gag and Staufen-1, we investigated if Staufen-2 and A3G can also interact with each other. Similar to the experiments performed to establish interactions between Staufen-2 and Staufen-1, immunoprecipitation assays using anti-HA affinity matrix in HEK293T cells expressing HA-tagged A3G and FLAG-tagged Staufen-2 were performed with FLAG-tagged vector as a negative control. Indeed, we detected Staufen-2 in A3G precipitates that were not affected by RNase A treatment or HIV-1 proviral DNA co-expression (Figure 5A). However, RNAse A treatment disrupted the A3G oligomerization, as described before [59] (Figure 5B). Staufen-2 interaction with A3G was also observed through confocal microscopy of co-expressing cells (Figure 5C). Interestingly, Staufen-2 co-localized within the RNPs carrying A3G, the association of which was found to be affected by RNase A treatment (Figure 5D). Fractions isolated by sucrose density gradient centrifugation of co-expressing cells suggested that a part of the Staufen-2 interaction with A3G is mediated by RNA, similar to, as reported earlier for Staufen-1 and A3G interaction [60,61]. RNase A treatment of RNP complexes majorly affects A3G and Staufen-1, but not Staufen-2 (Figure 4C and Figure 5D). This probably suggests an RNA-independent multimerization of Staufen-2 in cells, as well as a possibility of its co-association with other proteins in the RNP complex.

### 3.4. Fate of the Viral Population with Encapsidated Staufen-2 Alone or with Its Partners Staufen-1 or A3G

**Figure 6:**
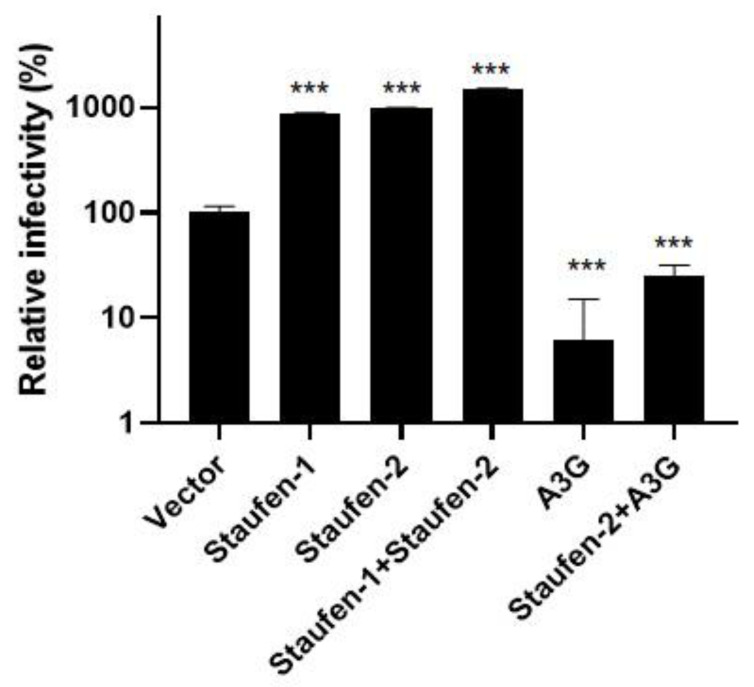
Impact of encapsidated Staufen-2 alone or with its partners Staufen-1 or A3G on viral infectivity: HIV-1Δ*vif* viral particles were produced from Staufen-2 FLAG or A3G HA or Staufen-1 FLAG, or vector alone transfected HEK293T cells. An equal amount of RT-activity normalized viral particles were used for the luciferase-based infectivity assay in HEK293T cells. Viral infectivity was quantified relative to the virus prepared from vector control cells. The presented values represent mean ± standard deviations (error bars) for three independent experiments. Asterisks (***) indicates *p*-value ≤ 0.0001.

Having confirmed that Staufen-2 can be encapsidated alone via interacting with HIV-1 Gag, we also observed Staufen-2 and Staufen-1 or A3G in the viral particles (speculatively indicating the chance of co-packaging of these proteins) (Supplementary Figure S4A,B). We were interested in testing the impact of HIV-1 particles produced by co-expressing Staufen-2 with a positive effector (Staufen-1) or with a negative effector (A3G). Equal amounts of RT-normalized viruses produced from HEK293T cells, either overexpressing Staufen-2 alone or co-overexpressing Staufen-2 and Staufen-1 or Staufen-2 and A3G, were used for luciferase-based infectivity assay as described above. We observed that packaging of Staufen-2 and Staufen-1 alone increased the viral infectivity. However, the co-packaging of Staufen-2 and Staufen-1 did not show an additive effect (Figure 6). Packaging of A3G in virus particles reduced the viral infectivity as reported [9,56], which could not be fully rescued by co-packaging Staufen-2 (Figure 6). This suggested the predominant impact of antiviral factor A3G on regulating HIV-1 propagation and infectivity compared to positive effectors Staufen-2 and Staufen-1.

## 4. Discussion

During the assembly and budding of HIV-1, several host factors remain associated with the newly emerging viruses [62,63,64]. These virus-associated or encapsidated host proteins may influence the infectivity of the virus population and decide the fate of disease progression [65,66,67]. Since Staufen-1, one such protein, is known to be packaged in several RNA viruses and acts as a positive effector for viral infectivity [12,14,35], here we comprehensively analyzed the viral packaging efficiency of Staufen-2, especially in HIV-1 for the first time. Herein, we report that Staufen-2 expressed in virus producer cells is efficiently incorporated into HIV-1 particles potentially via Gag interaction and promotes their infectivity. This expands our knowledge of different Staufen proteins’ function in HIV-1 replication. Our previous study identified Staufen-2 as a host factor that is up-regulated in Sup-T1 and likely in other cells during HIV-1 infection. It can enhance virus production by directly interacting with Rev and facilitating its RNA export activity [40]. Here, we essentially investigated its protein binding features and confirmed that HIV-1 Gag, Staufen-1, and A3G are indeed some interacting partners.

While the packaging of restriction factors such as APOBEC3 proteins into virions is a prerequisite for targeting the early post-entry phase of infection, it is interesting to note the packaging of dependency factors such as Staufen proteins, which work in favor of HIV-1 production in both producer and target cells. The RNA- as well as Gag-interactome, is conceivably complex. Therefore, it is possible to have several nonspecific RNA–protein and protein–protein molecular interactions during HIV-1 assembly, ultimately carrying them into viral particles during egression. Therefore, we created Staufen-2 knockout cells to understand their influence on viral infectivity and production. The results conclusively demonstrate that the absence of Staufen-2 (to a lesser extent, Staufen-1 too) impairs the virion production and their infectivity. This highlights a new role of virion-bound Staufen proteins in promoting HIV-1 infection, likely during early post-entry events. It was also interesting to observe that Staufen-2, when packaged in an HIV-1Δ*vif*, caused increased infectivity (Figure 6), a finding that may justify further investigation.

We observed the packaging of Staufen-2 into SIVagm virions (Supplementary Figure S5), which suggests that encapsidation of RNA-binding proteins may be a more fundamental phenomenon. This raises the likelihood of Staufen-2′s interaction with other retroviruses such as murine leukemia virus (MLV) and other RNA viruses, although this remains to be elucidated. Our results can only postulate that the mechanism(s) that transports cellular Staufen-2 to HIV-1 and SIVagm are likely to be similar.

According to the BioGRID database (thebiogrid.org/117978, which maintains curated entries of biological interaction based on original publications), there are 108 protein interactors of Staufen-2, including Staufen-1, MOV10 RNA helicase, HIV-1 Tat, ADAR RNA adenosine deaminase, and nucleocapsid of SARS-CoV-2. In addition, a previous study using mass spectrometry identified 45 proteins (22%) from Staufen-1 associated RNPs, which were found in HIV-1 particles, suggesting that RNP complexes could likely serve as vehicles [61]. These data prompted us to study Staufen-2 interaction with the host and HIV-1 proteins, and we included HIV-1 Gag, Staufen-1, and A3G to explore the underlying modes of viral packaging. As anticipated, Staufen-2 interacted with Gag and is probably responsible for its packaging into HIV-1. Additionally, its interaction with other host proteins such as Staufen-1 and A3G, characterized as RNP complex components, is consistent. This evidence reports an independent binding of Staufen-2 with Staufen-1 and A3G, which hypothetically raises the possibility of different modes of viral incorporation and, consequently, their synergetic action in HIV-1 replication. Certainly, follow-up studies using single virion microscopy to determine co-packaging rates and detailed mutational analysis to identify responsible residues mediating interactions may aid in their validation. Our results propose a model in which Staufen-2 packaging into HIV-1 is mediated by its interaction with Gag and probably independent binding of factors such as a positive effector, Staufen-1, or a negative (restriction) effector, A3G, collectively accelerates its viral encapsidation.

Since the distribution of host factors during packaging is dynamic, different virions emerging from an infected cell are likely to carry varying compositions of host protein contributing to a pool of proteomically divergent virions (quasispecies) [68,69,70]. This differential host protein packaging may explain the basis of differential infectivity of emerging virions from different cell types, cellular microenvironment, factors that stimulate restriction factors (cytokine environment of the infected cell), depleting proviral factors, which together would decide the fate of viral infectivity and disease progression. Hence, the viral populations studied here would be expected to contain differential amounts of packaged proteins in diverse combinations such as Staufen-2 only, or Staufen-2 associated with Staufen-1/A3G, or either of one factor or even none. This perspective also thrusts an evolutionary advantage to the viral population carrying predominantly positive effectors such as Staufen and disadvantage to the viral population carrying predominantly negative effectors such as A3G. Overall, these factors can drive the viral population dynamics, infectivity, and evolution within a patient deciding the fate of disease progression.

Moreover, there is a likelihood of involvement of Staufen-2 in Staufen-1-mediated processes during HIV infection, such as disruption of stress granule formation [12,13]. The increase in the infectivity of the viral population with encapsidated Staufen-2 may be speculated due to increased protection provided to HIV-1 RNA against RNA recognition and degradation by host sensors. In addition, conceptually there is a possibility of Staufen-2′s function in nuclear import, and RT. The additional roles of Staufen-2 may be clarified by identifying Staufen-2 residing viral compartment. Further, as phenylalanine glycine motif (FG-motif) containing proteins, such as Sec24C, tends to increase the HIV-1 capsid stability [71], FG-motif present in Staufen-2 RNA-Binding-Domain 2 (Uniprot Q9NUL3) [19] may also function similarly, contributing to increased viral infectivity.

It is interesting to note that while packaging of Staufen-2 enhances the viral infectivity, HIV-1 produced from cells expressing A3G/Staufen-2, the infectivity of the viral population is reduced (Figure 6). The result indicates that the antiviral activity of A3G could not be entirely overcome by either Staufen-2 alone or Staufen-2 interacting with A3G. Similarly, we also expected that Staufen-2 would not have any inhibitory effect on either the stability or enzymatic activity of A3G. Our study supports that Staufen-2 neither protects A3G from Vif-mediated degradation (Supplementary Figure S6A–C) nor inhibits the cytidine deaminase activity of A3G (Supplementary Figure S6D,E). These observations rationalize why the antiviral impact of A3G prevails, even when likely packaged with Staufen-2 (Figure 6). The interaction of Staufen-2 with Staufen-1 had no added effect on the infectivity of the virions.

## 5. Conclusions

Overall, our study dissects the mechanistic details of Staufen-2 incorporation into emerging virus particles from producer cells, wherein we identified that the packaging of Staufen-2 boosts the infectivity of HIV-1. In conclusion, this study offers new perspectives on the co-evolution of host–pathogen interactions, where HIV-1 adopts the multifunctional Staufen proteins to augment its propagation and increase the infectivity of the emerging viral population in an infected host, thereby influencing disease prognosis.

## Data Availability

All relevant data can be found in the manuscript and supporting information files.

## Supplementary Materials

The following are available online at https://www.mdpi.com/article/10.3390/v13122459/s1, Figure S1: HIV-1 viral core isolation to evaluate Staufen-2 encapsidation, Figure S2: Generation of Staufen-2 and Staufen-1 KO cell lines and their infectivity, Figure S3: Staufen-2 and Staufen-1 interaction, Figure S4: Staufen-2 is incorporated into HIV-1 with other cellular factors, Figure S5: Staufen-2 is incorporated into SIVagm virions, Figure S6: Staufen-2 does not disrupt Vif-mediated A3G degradation or A3G cytidine deaminase activity.

## References

[1] Cochrane A.W., McNally M.T., Mouland A.J. (2006). The retrovirus RNA trafficking granule: From birth to maturity. Retrovirology.

[2] Knoener R.A., Becker J.T., Scalf M., Sherer N.M., Smith L.M. (2017). Elucidating the in vivo interactome of HIV-1 RNA by hybridization capture and mass spectrometry. Sci. Rep..

[3] Garcia-Moreno M., Järvelin A.I., Castello A. (2018). Unconventional RNA-binding proteins step into the virus–host battlefront. Wiley Interdiscip. Rev. RNA.

[4] Freed E.O. (1998). HIV-1 Gag Proteins: Diverse Functions in the Virus Life Cycle. Virology.

[5] Tuffy K.M., Maldonado R.J.K., Chang J., Rosenfeld P., Cochrane A., Parent L.J. (2020). HIV-1 Gag Forms Ribonucleoprotein Complexes with Unspliced Viral RNA at Transcription Sites. Viruses.

[6] Xu W., Byun H., Dudley J. (2020). The Role of APOBECs in Viral Replication. Microorganisms.

[7] Harris R.S., Dudley J.P. (2015). APOBECs and virus restriction. Virology.

[8] Browne E.P., Allers C., Landau N.R. (2009). Restriction of HIV-1 by APOBEC3G is cytidine deaminase-dependent. Virology.

[9] Soros V.B., Yonemoto W., Greene W.C. (2007). Newly Synthesized APOBEC3G Is Incorporated into HIV Virions, Inhibited by HIV RNA, and Subsequently Activated by RNase H. PLoS Pathog..

[10] Chiu Y.-L., Soros V.B., Kreisberg J.F., Stopak K., Yonemoto W., Greene W.C. (2005). Cellular APOBEC3G restricts HIV-1 infection in resting CD4+ T cells. Nature.

[11] Chatel-Chaix L., Boulay K., Mouland A.J., DesGroseillers L. (2008). The host protein Staufen1 interacts with the Pr55Gagzinc fingers and regulates HIV-1 assembly via its N-terminus. Retrovirology.

[12] Rao S., Hassine S., Monette A., Amorim R., DesGroseillers L., Mouland A.J. (2019). HIV-1 requires Staufen1 to dissociate stress granules and to produce infectious viral particles. RNA.

[13] Abrahamyan L.G., Chatel-Chaix L., Ajamian L., Milev M.P., Monette A., Clément J.-F., Song R., Lehmann M., DesGroseillers L., Laughrea M. (2010). Novel Staufen1 ribonucleoproteins prevent formation of stress granules but favour encapsidation of HIV-1 genomic RNA. J. Cell Sci..

[14] Mouland A.J., Mercier J., Luo M., Bernier L., DesGroseillers L., Cohen E.A. (2000). The Double-Stranded RNA-Binding Protein Staufen Is Incorporated in Human Immunodeficiency Virus Type 1: Evidence for a Role in Genomic RNA Encapsidation. J. Virol..

[15] Heraud-Farlow J., Kiebler M.A. (2014). The multifunctional Staufen proteins: Conserved roles from neurogenesis to synaptic plasticity. Trends Neurosci..

[16] Park E., Maquat L.E. (2013). Staufen-mediated mRNA decay. Wiley Interdiscip. Rev. RNA.

[17] Miki T., Takano K., Yoneda Y. (2005). The Role of Mammalian Staufen on mRNA Traffic: A View from Its Nucleocytoplasmic Shuttling Function. Cell Struct. Funct..

[18] Furic L., Maher-Laporte M., DesGroseillers L. (2007). A genome-wide approach identifies distinct but overlapping subsets of cellular mRNAs associated with Staufen1- and Staufen2-containing ribonucleoprotein complexes. RNA.

[19] Heber S., Gáspár I., Tants J.-N., Günther J., Moya S.M.F., Janowski R., Ephrussi A., Sattler M., Niessing D. (2019). Staufen2-mediated RNA recognition and localization requires combinatorial action of multiple domains. Nat. Commun..

[20] Lebeau G., Miller L.C., Tartas M., McAdam R., Laplante I., Badeaux F., DesGroseillers L., Sossin W.S., Lacaille J.-C. (2011). Staufen 2 regulates mGluR long-term depression and Map1b mRNA distribution in hippocampal neurons. Learn. Mem..

[21] Macchi P., Brownawell A.M., Grunewald B., DesGroseillers L., Macara I.G., Kiebler M. (2004). The Brain-specific Double-stranded RNA-binding Protein Staufen2. J. Biol. Chem..

[22] Park E., Gleghorn M.L., Maquat L.E. (2012). Staufen2 functions in Staufen1-mediated mRNA decay by binding to itself and its paralog and promoting UPF1 helicase but not ATPase activity. Proc. Natl. Acad. Sci. USA.

[23] Goetze B., Tuebing F., Xie Y., Dorostkar M., Thomas S., Pehl U., Boehm S., Macchi P., Kiebler M. (2006). The brain-specific double-stranded RNA-binding protein Staufen2 is required for dendritic spine morphogenesis. J. Cell Biol..

[24] Zhong Y., Hu Z., Wu J., Dai F., Lee F., Xu Y. (2020). STAU1 selectively regulates the expression of inflammatory and immune response genes and alternative splicing of the nerve growth factor receptor signaling pathway. Oncol. Rep..

[25] Yoon J.-S., Mogilicherla K., Gurusamy D., Chen X., Chereddy S., Palli S.R. (2018). Double-stranded RNA binding protein, Staufen, is required for the initiation of RNAi in coleopteran insects. Proc. Natl. Acad. Sci. USA.

[26] Bilogan C.K., Horb M.E. (2011). Xenopus staufen2 is required for anterior endodermal organ formation. Genesis.

[27] Zhang X., Trépanier V., Beaujois R., Viranaicken W., Drobetsky E., DesGroseillers L. (2016). The downregulation of the RNA-binding protein Staufen2 in response to DNA damage promotes apoptosis. Nucleic Acids Res..

[28] Condé L., Quesada Y.G., Bonnet-Magnaval F., Beaujois R., DesGroseillers L. (2021). STAU2 protein level is controlled by caspases and the CHK1 pathway and regulates cell cycle progression in the non-transformed hTERT-RPE1 cells. BMC Mol. Cell Biol..

[29] Cockburn D.M., Charish J., Tassew N.G., Eubanks J., Bremner R., Macchi P., Monnier P.P. (2012). The double-stranded RNA-binding protein Staufen 2 regulates eye size. Mol. Cell. Neurosci..

[30] Chen Y.-M., Ou B.-T., Chen C.-Y., Chan H.-H., Chen C.-J., Wang R.Y. (2019). Staufen1 Protein Participates Positively in the Viral RNA Replication of Enterovirus 71. Viruses.

[31] de Lucas S., Peredo J., Marión R.M., Sánchez C., Ortín J. (2010). Human Staufen1 Protein Interacts with Influenza Virus Ribonucleoproteins and Is Required for Efficient Virus Multiplication. J. Virol..

[32] Blackham S.L., McGarvey M. (2013). A host cell RNA-binding protein, Staufen1, has a role in hepatitis C virus replication before virus assembly. J. Gen. Virol..

[33] Dixit U., Pandey A.K., Mishra P., Sengupta A., Pandey V.N. (2016). Staufen1 promotes HCV replication by inhibiting protein kinase R and transporting viral RNA to the site of translation and replication in the cells. Nucleic Acids Res..

[34] Hanke K., Hohn O., Liedgens L., Fiddeke K., Wamara J., Kurth R., Bannert N. (2013). Staufen-1 Interacts with the Human Endogenous Retrovirus Family HERV-K(HML-2) Rec and Gag Proteins and Increases Virion Production. J. Virol..

[35] Chatel-Chaix L., Clément J.-F., Martel C., Bériault V., Gatignol A., DesGroseillers L., Mouland A.J. (2004). Identification of Staufen in the Human Immunodeficiency Virus Type 1 Gag Ribonucleoprotein Complex and a Role in Generating Infectious Viral Particles. Mol. Cell. Biol..

[36] Chatel-Chaix L., Abrahamyan L., Fréchina C., Mouland A.J., DesGroseillers L. (2007). The Host Protein Staufen1 Participates in Human Immunodeficiency Virus Type 1 Assembly in Live Cells by Influencing pr55 Gag Multimerization. J. Virol..

[37] Bélanger G., Stocksley M.A., Vandromme M., Schaeffer L., Furic L., DesGroseillers L., Jasmin B.J. (2003). Localization of the RNA-binding proteins Staufen1 and Staufen2 at the mammalian neuromuscular junction. J. Neurochem..

[38] Mallardo M., Deitinghoff A., Müller J., Goetze B., Macchi P., Peters C., Kiebler M.A. (2003). Isolation and characterization of Staufen-containing ribonucleoprotein particles from rat brain. Proc. Natl. Acad. Sci. USA.

[39] Thomas M.G., Tosar L.J.M., Loschi M., Pasquini J.M., Correale J., Kindler S., Boccaccio G.L. (2005). Staufen Recruitment into Stress Granules Does Not Affect Early mRNA Transport in Oligodendrocytes. Mol. Biol. Cell.

[40] Banerjee A., Benjamin R., Balakrishnan K., Ghosh P., Banerjee S. (2014). Human protein Staufen-2 promotes HIV-1 proliferation by positively regulating RNA export activity of viral protein Rev. Retrovirology.

[41] Marin M., Rose K.M., Kozak S.L., Kabat D. (2003). HIV-1 Vif protein binds the editing enzyme APOBEC3G and induces its degradation. Nat. Med..

[42] Sugimoto Y., Vigilante A., Darbo E., Zirra A., Militti C., D’Ambrogio A., Luscombe N.M., Ule J. (2015). hiCLIP reveals the in vivo atlas of mRNA secondary structures recognized by Staufen 1. Nature.

[43] Russell R.A., Wiegand H.L., Moore R., Schäfer A., McClure M.O., Cullen B.R. (2005). Foamy Virus Bet Proteins Function as Novel Inhibitors of the APOBEC3 Family of Innate Antiretroviral Defense Factors. J. Virol..

[44] Sanjana N.E., Shalem O., Zhang F. (2014). Improved vectors and genome-wide libraries for CRISPR screening. Nat. Methods.

[45] Shalem O., Sanjana N.E., Hartenian E., Shi X., Scott D.A., Mikkelsen T.S., Heckl D., Ebert B.L., Root D.E., Doench J.G. (2014). Genome-Scale CRISPR-Cas9 Knockout Screening in Human Cells. Science.

[46] Sunseri N., O’Brien M., Bhardwaj N., Landau N.R. (2011). Human Immunodeficiency Virus Type 1 Modified to Package Simian Immunodeficiency Virus Vpx Efficiently Infects Macrophages and Dendritic Cells. J. Virol..

[47] Dull T., Zufferey R., Kelly M., Mandel R.J., Nguyen M., Trono D., Naldini L. (1998). A Third-Generation Lentivirus Vector with a Conditional Packaging System. J. Virol..

[48] Srivastava K.K., Fernandez-Larsson R., Zinkus D.M., Robinson H.L. (1991). Human immunodeficiency virus type 1 NL4-3 replication in four T-cell lines: Rate and efficiency of entry, a major determinant of permissiveness. J. Virol..

[49] Mariani R., Chen D., Schröfelbauer B., Navarro F., König R., Bollman B., Münk C., Nymark-McMahon H., Landau N.R. (2003). Species-Specific Exclusion of APOBEC3G from HIV-1 Virions by Vif. Cell.

[50] Vasudevan A.A.J., Hofmann H., Willbold D., Häussinger D., Koenig B., Münk C. (2017). Enhancing the Catalytic Deamination Activity of APOBEC3C Is Insufficient to Inhibit Vif-Deficient HIV-1. J. Mol. Biol..

[51] Xiao X., Li S.-X., Yang H., Chen X.S. (2016). Crystal structures of APOBEC3G N-domain alone and its complex with DNA. Nat. Commun..

[52] Wang X., Dolan P.T., Dang Y., Zheng Y.-H. (2007). Biochemical Differentiation of APOBEC3F and APOBEC3G Proteins Associated with HIV-1 Life Cycle. J. Biol. Chem..

[53] Vasudevan A.A.J., Balakrishnan K., Gertzen C.G.W., Borvető F., Zhang Z., Sangwiman A., Held U., Küstermann C., Banerjee S., Schumann G.G. (2020). Loop 1 of APOBEC3C Regulates its Antiviral Activity against HIV-1. J. Mol. Biol..

[54] Marino D., Perković M., Hain A., Vasudevan A.A.J., Hofmann H., Hanschmann K.-M., Mühlebach M.D., Schumann G.G., König R., Cichutek K. (2016). APOBEC4 Enhances the Replication of HIV-1. PLoS ONE.

[55] Kuffour E.O., Schott K., Vasudevan A.A.J., Holler J., Schulz W.A., Lang P.A., Lang K.S., Kim B., Häussinger D., König R. (2018). USP18 (UBP43) Abrogates p21-Mediated Inhibition of HIV-1. J. Virol..

[56] Vasudevan A.A.J., Perković M., Bulliard Y., Cichutek K., Trono D., Häussinger D., Münk C. (2013). Prototype Foamy Virus Bet Impairs the Dimerization and Cytosolic Solubility of Human APOBEC3G. J. Virol..

[57] Draper D.E., Reynaldo L.P. (1999). RNA binding strategies of ribosomal proteins. Nucleic Acids Res..

[58] Horn A.V., Klawitter S., Held U., Berger A., Vasudevan A.A.J., Bock A., Hofmann H., Hanschmann K.-M.O., Trösemeier J.-H., Flory E. (2013). Human LINE-1 restriction by APOBEC3C is deaminase independent and mediated by an ORF1p interaction that affects LINE reverse transcriptase activity. Nucleic Acids Res..

[59] Huthoff H., Autore F., Gallois-Montbrun S., Fraternali F., Malim M.H. (2009). RNA-Dependent Oligomerization of APOBEC3G Is Required for Restriction of HIV-1. PLoS Pathog..

[60] Gallois-Montbrun S., Kramer B., Swanson C., Byers H., Lynham S., Ward M., Malim M.H. (2007). Antiviral Protein APOBEC3G Localizes to Ribonucleoprotein Complexes Found in P Bodies and Stress Granules. J. Virol..

[61] Milev M.P., Ravichandran M., Khan M.F., Schriemer D.C., Mouland A.J. (2012). Characterization of Staufen1 Ribonucleoproteins by Mass Spectrometry and Biochemical Analyses Reveal the Presence of Diverse Host Proteins Associated with Human Immunodeficiency Virus Type 1. Front. Microbiol..

[62] Sundquist W.I., Kräusslich H.-G. (2012). HIV-1 Assembly, Budding, and Maturation. Cold Spring Harb. Perspect. Med..

[63] Freed E.O. (2015). HIV-1 assembly, release and maturation. Nat. Rev. Genet..

[64] Ramdas P., Sahu A.K., Mishra T., Bhardwaj V., Chande A. (2020). From Entry to Egress: Strategic Exploitation of the Cellular Processes by HIV-1. Front. Microbiol..

[65] Colomer-Lluch M., Ruiz A., Moris A., Prado J.G. (2018). Restriction Factors: From Intrinsic Viral Restriction to Shaping Cellular Immunity Against HIV-1. Front. Immunol..

[66] Temple J., Tripler T.N., Shen Q., Xiong Y. (2020). A snapshot of HIV-1 capsid–host interactions. Curr. Res. Struct. Biol..

[67] Lama J., Planelles V. (2007). Host factors influencing susceptibility to HIV infection and AIDS progression. Retrovirology.

[68] Ode H., Matsuda M., Matsuoka K., Hachiya A., Hattori J., Kito Y., Yokomaku Y., Iwatani Y., Sugiura W. (2015). Quasispecies Analyses of the HIV-1 Near-full-length Genome with Illumina MiSeq. Front. Microbiol..

[69] De Azevedo S.S.D., Caetano D.G., Côrtes F.H., Teixeira S.L.M., Silva K.D.S., Hoagland B., Grinsztejn B., Veloso V.G., Morgado M.G., Bello G. (2017). Highly divergent patterns of genetic diversity and evolution in proviral quasispecies from HIV controllers. Retrovirology.

[70] Sguanci L., Bagnoli F., Liò P. (2007). Modeling HIV quasispecies evolutionary dynamics. BMC Evol. Biol..

[71] Rebensburg S.V., Wei G., Larue R.C., Lindenberger J., Francis A.C., Annamalai A.S., Morrison J., Shkriabai N., Huang S.-W., KewalRamani V. (2021). Sec24C is an HIV-1 host dependency factor crucial for virus replication. Nat. Microbiol..

