# Supplementary material for: Encapsidation of Staufen-2 Enhances Infectivity of HIV-1
Source: Viruses. 2021 Dec 8;13(12):2459. doi: 10.3390/v13122459 (PMC8703407; doi:10.3390/v13122459)
Supplement: Supplementary file 1 [file viruses-13-02459-s001.zip › Supplementary Material viruses-1399737_To be Published Final version.pdf]

Encapsidation of Staufen-2 enhances infectivity of HIV-1

Kannan Balakrishnan [1,2], Ananda Ayyappan Jaguva Vasudevan [2,†,‡], Krishnaveni Mohareer [1,‡], Tom Luedde [2], Carsten Münk [2,*], Sharmistha Banerjee [1,*]

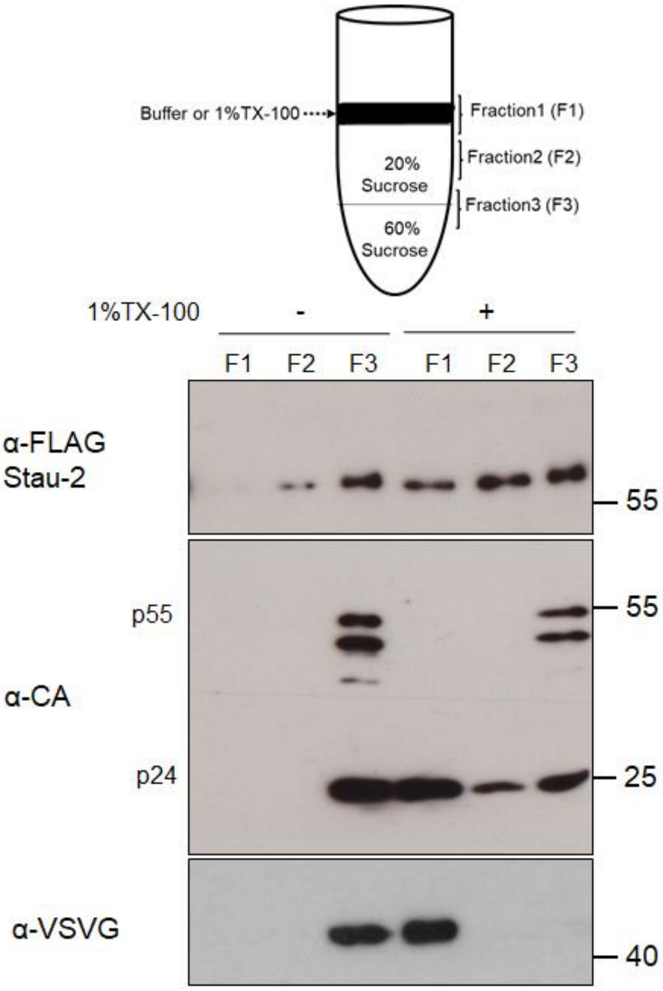

Figure S1: HIV-1 viral core isolation to evaluate Staufen-2 encapsidation: HIV-1Δ*vif* virions were produced in the presence of Staufen-2, and the viral cores were isolated by using 20%/60% sucrose step gradient centrifugation, wherein 1% triton X-100 was overlaid on the top as described earlier (Jaguva Vasudevan et al. 2017a) (see methods). As shown in the schematic, three fractions F1, F2, and F3 were collected from the top of the sucrose step gradient. F1 fraction contains soluble proteins; F2 fraction is a buffer fraction of 20% sucrose that separates soluble proteins from virus particles or viral cores; F3 fraction includes the interphase of 20%/60% sucrose viral particles (not TX-100), and viral cores (TX-100) accumulate. An aliquot of sucrose step gradient fractions was subjected to Western analysis to detect HIV capsids (α-p24), Staufen-2 (α-FLAG), and VSVG (α-VSVG) with respective antibodies. TX-100 represents Triton X-100.

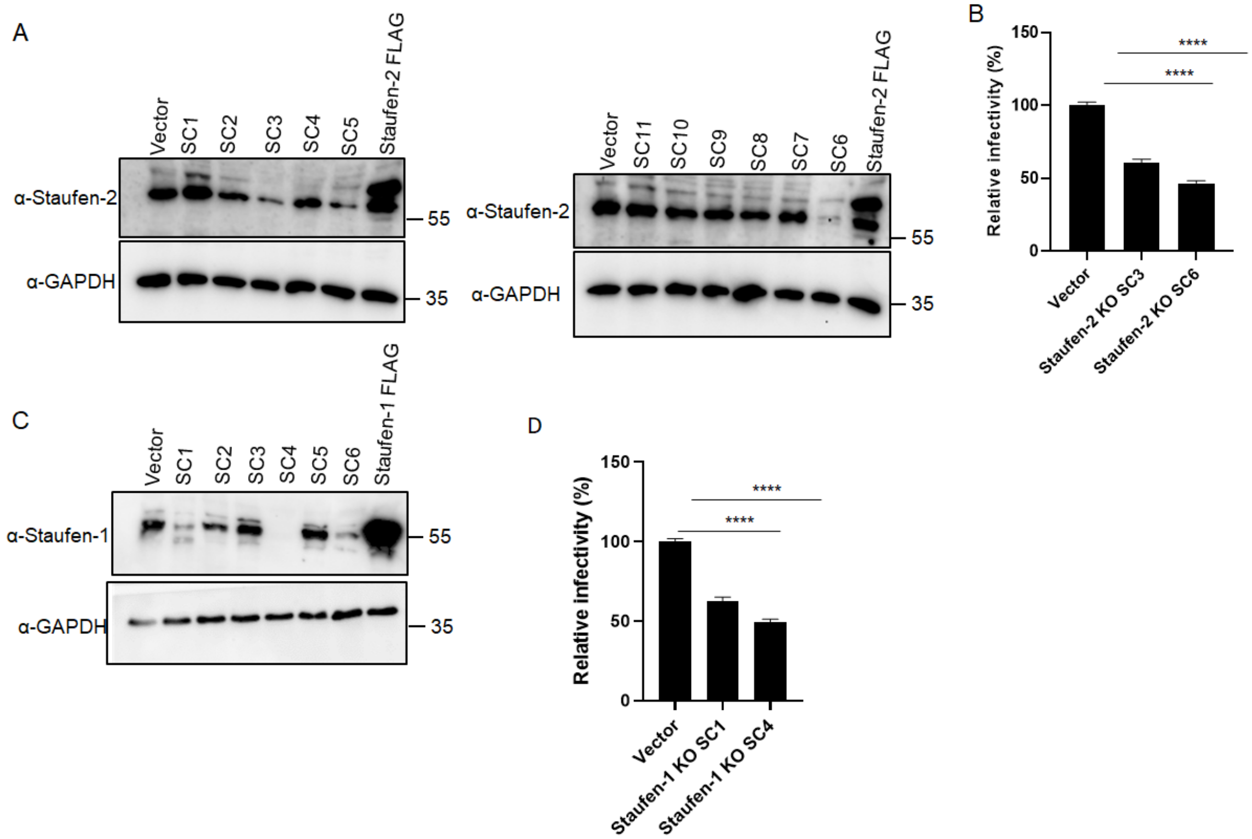

Figure S2: Generation of Staufen-2 and Staufen-1 KO cell lines and their infectivity: (A) Staufen-2 KO was generated in HEK293T cells using CRISPR-Cas9 as described in the methods. To confirm the knockout of Staufen-2 expression, cell lysates of single clones of both vector and Staufen-2 KO were probed with Western analyses using an anti-Staufen-2 antibody. The absence of a specific band in clone 6 confirms Staufen-2 KO. **(B)** An equal amount of RT-activity normalized viral particles were used for the luciferase-based infectivity assay in vector cells and Staufen-2 KO single clones 3 and 6. **(C)** Similarly, Staufen-1 KO was generated in HEK293T cells and **(D)** Luciferase-based -infectivity assays were performed in vector cells and Staufen-1 KO single clones 1 and 4. Viral infectivity was quantified relative to the virus infected with vector control. The presented values represent mean ± standard deviations (error bars) for three independent experiments. Asterisks (****) indicates p-value ≤ 0.0001.

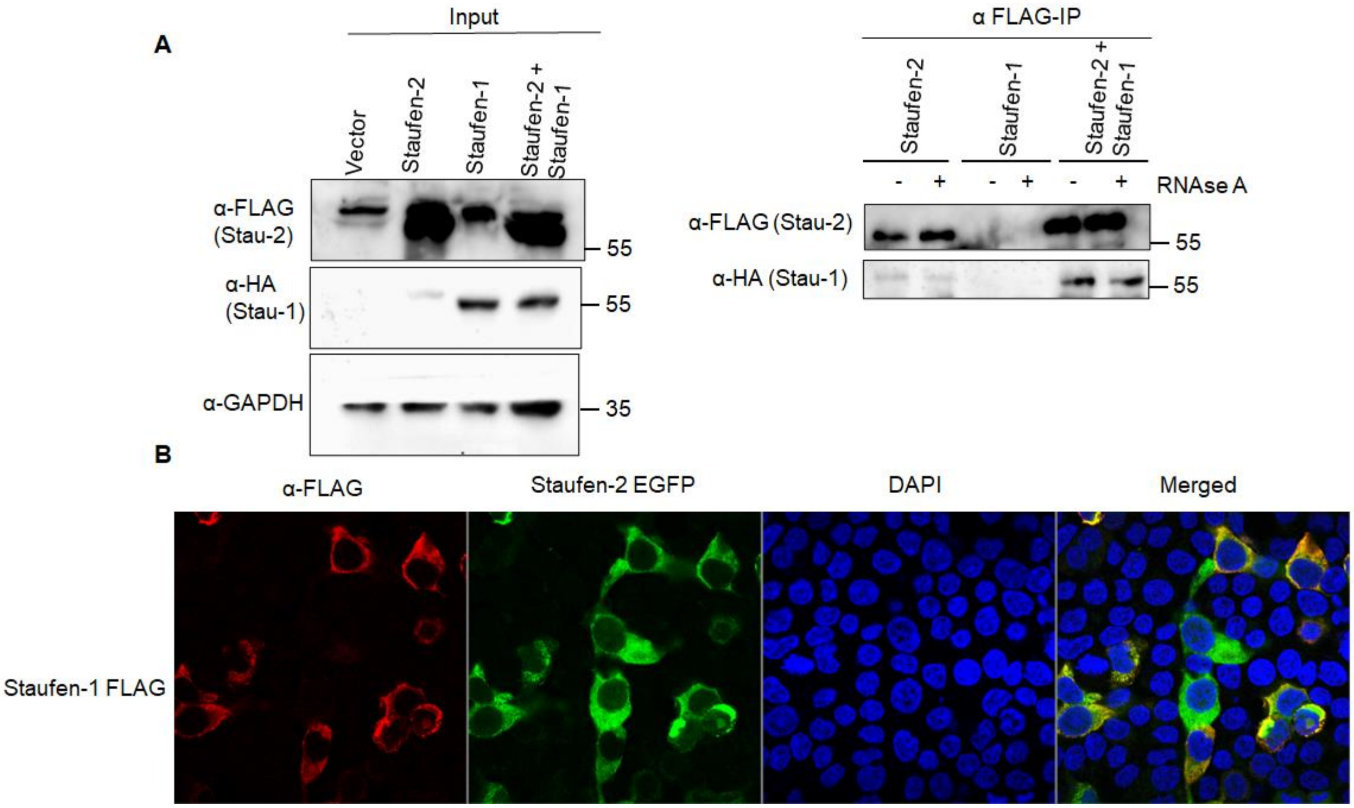

Figure S3: Staufen-2 and Staufen-1 interaction: (A) HEK293T cells were transfected with Staufen-2 FLAG or Staufen-1 HA, or both followed by IP with anti-FLAG beads and the interactions were confirmed by Western analyses **(B)** HEK293T cells were transfected with Staufen-1 FLAG and Staufen-2 EGFP N1 and monitored for their co-localization.

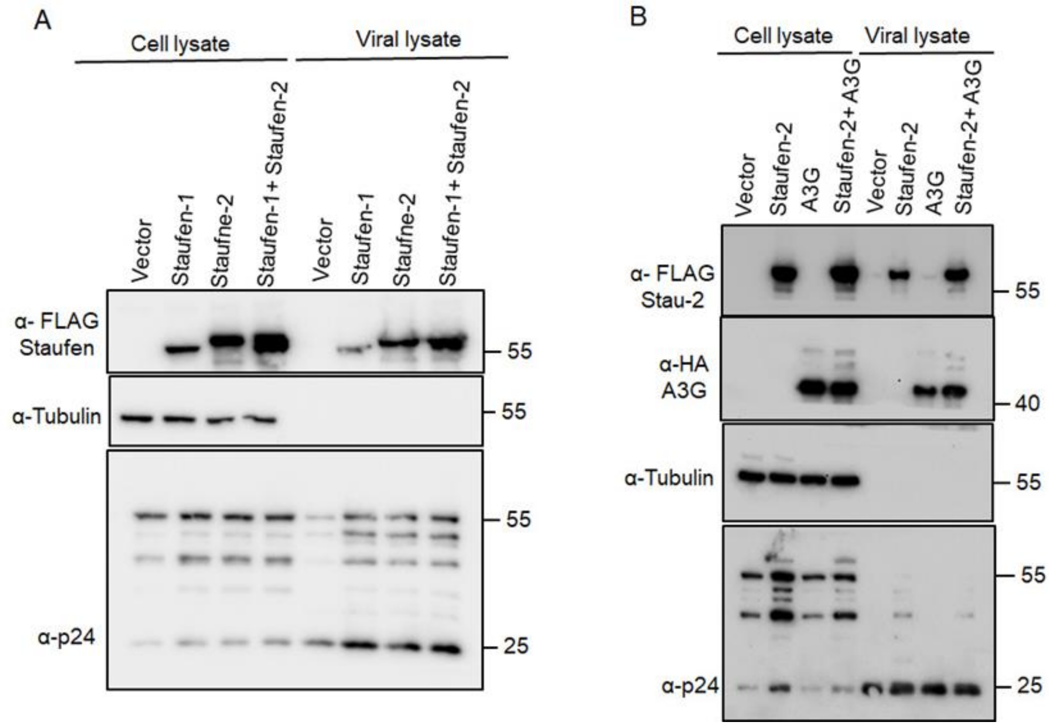

Figure S4: Staufen-2 is incorporated into HIV-1 with other cellular factors: (A) HIV-1Δ*vif* viral particles were produced from HEK293T cells transfected with Staufen-2 FLAG or Staufen-1 FLAG, or both constructs. Vector alone transfected HEK293T cells were used as a control. Western analyses were carried out using anti-FLAG (Staufen-2 and Staufen-1) and anti-p24 antibodies (HIV-1 capsid protein) in the cell and viral lysates. **(B)** HIV-1Δ*vif* viral particles were produced from HEK293T cells transfected with Staufen-2 FLAG or A3G HA or both constructs or vector alone. Western analyses were carried out using anti-FLAG (Staufen-2) and anti-HA (A3G HA) anti-p24 antibodies in the cell and viral lysate. Tubulin was used as a loading control.

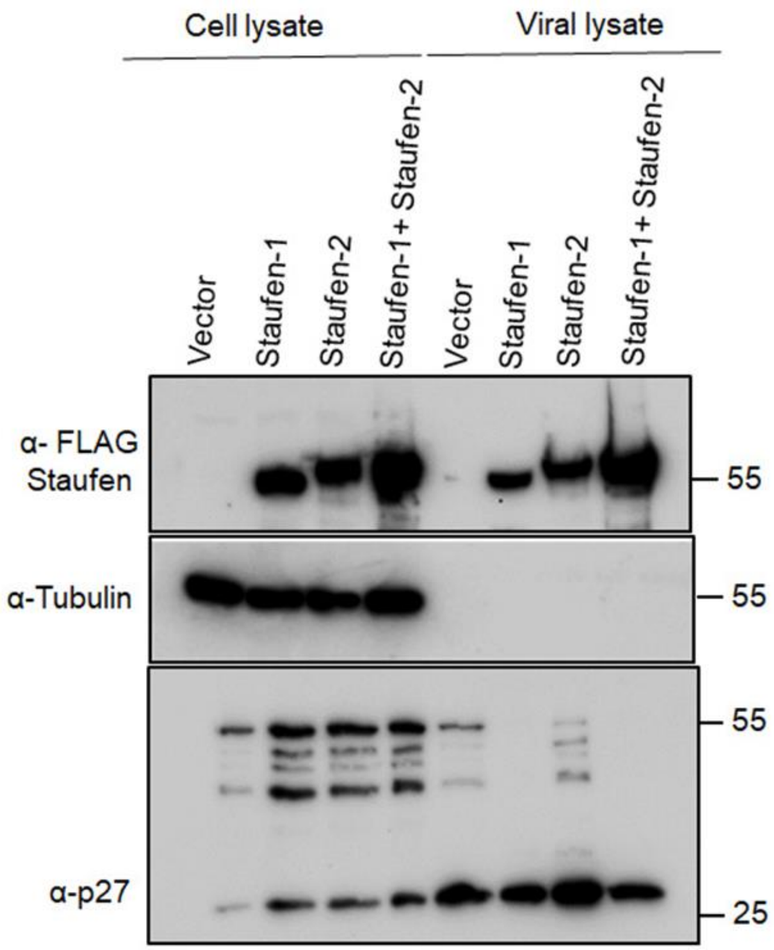

Figure S5: Staufen-2 is incorporated into SIVagm virions: SIV-1Δ*vif* viral particles were produced from HEK293T cells transfected with Staufen-2 FLAG or Staufen-1 FLAG, or both constructs or vector-only. Western blotting analyses were carried out using anti-FLAG (Staufen-2 and Staufen-1) and anti-p24 antibodies (detecting SIV-1 capsid protein) in the cell and viral lysates with Tubulin as a loading control.

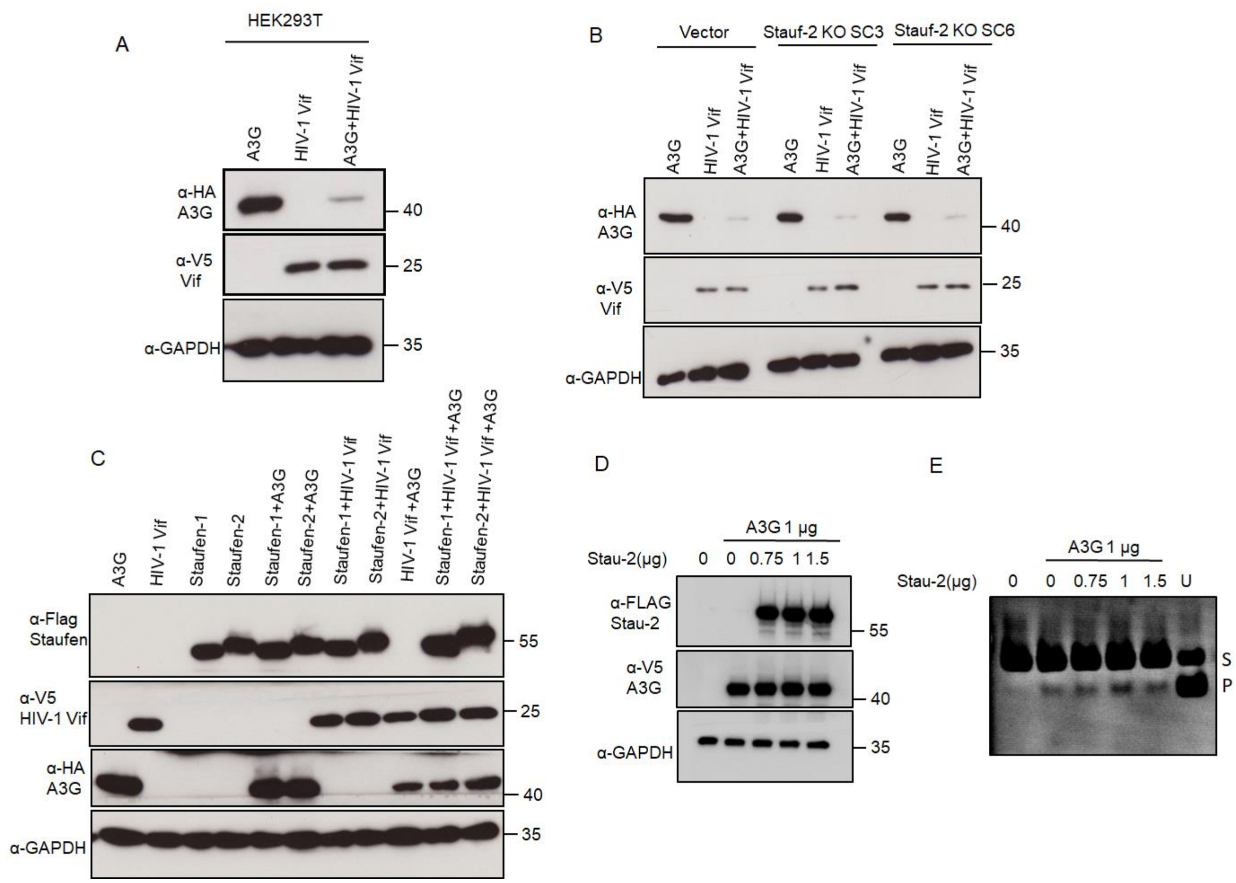

Figure S6: Staufen-2 does not disrupt Vif-mediated A3G degradation or A3G cytidine deaminase activity: (A) HEK293T cells were co-transfected with A3G HA-tagged construct and HIV-1 Vif V5 construct. The expression of individual proteins was assessed by Western analyses using respective antibodies. (**B)** Similarly, HEK293T vector and Staufen-2 KO cells were co-transfected with A3G HA-tagged construct and HIV-1 Vif-V5 construct. (**C)** HEK293T cells were co-transfected with either A3G HA-tagged construct along with HIV-1 Vif V5 construct or Staufen-2 or Staufen-1. **(D)** HEK293T cells were transfected with vector alone or A3G V5, A3G V5 with increasing concentration of Staufen-2 FLAG expression plasmid. The expression of individual proteins was assessed by Western analyses using respective antibodies. GAPDH served as a loading control in all Western analyses. **(E)** *In vitro* cytidine deamination activity of A3G upon Staufen-2 overexpression. Samples were treated with RNase A, and oligonucleotide-containing uracil (U) instead of cytosine served as a marker to denote the migration of deaminated product after restriction enzyme cleavage. S-substrate, P-product.
